# In Vitro Molecular Study of Titanium-Niobium Alloy Biocompatibility

**DOI:** 10.3390/biomedicines10081898

**Published:** 2022-08-05

**Authors:** Laëtitia Chézeau, Alex Tchinda, Gaël Pierson, Pierre Bravetti, Luc Ferrari, Olivier Joubert, Mohamed Zaiou, Bertrand H. Rihn

**Affiliations:** Institut Jean Lamour, University of Lorraine, UMR CNRS 7198, 54011 Nancy, France

**Keywords:** titanium, niobium, potassium niobate, dental implant, transcriptome, biocompatible materials, cytotoxicity, immunotoxicity, monocyte, osteoblast

## Abstract

Titanium dental implants have common clinical applications due to their biocompatibility, biophysical and biochemical characteristics. Although current titanium is thought to be safe and beneficial for patients, there are several indications that it may release toxic metal ions or metal nanoparticles from its alloys into the surrounding environment, which could lead to clinically relevant complications including toxic reactions as well as immune dysfunctions. Hence, an adequate selection and testing of medical biomaterial with outstanding properties are warranted. This study was designed to explore the biocompatibility of smooth titanium-niobium alloy (S_TiNb) versus smooth titanium commercially pure (S_TiCp)—a reference in implantology. All experiments were performed in vitro using human osteoblast-like SaOs-2 and monocyte THP-1 cell lines as models. Cell adhesion and growth morphology were determined by scanning electron microscopy, while cell viability was evaluated using WST-1 assay. Because niobate anions or niobium nanoparticles can be released from implants during biomaterial-cell interaction, potential immunotoxicity of potassium niobate (KNbO_3)_ salt was evaluated by examining both metabolic activity and transcriptomic profiling of treated THP-1 monocytes. The main findings of this study are that S_TiCp and S_TiNb discs do not show an impact on the proliferation and viability of SaOs-2 cells compared to polystyrene surfaces, whereas a significant decrease in THP-1 cells’ viability and metabolic activity was observed in the presence of S_TiNb discs compared to the control group. However, no significant changes were found neither at the metabolic activity nor at the transcriptomic level of THP-1 monocytes exposed to KNbO_3_ salt, suggesting that niobium has no effect on the immune system. Overall, these data imply a possible toxicity of S_TiNb discs toward THP-1 cells, which may not be directly related to niobium but perhaps to the manufacturing process of titanium-niobium alloy. Thus, this limitation must be overcome to make titanium alloy an excellent material for medical applications.

## 1. Introduction

Dental implants are reference treatment for replacing missing teeth. The first attempts to implant teeth go back to the days of the ancient Egyptians in 2500 BC [1]. However, truly healthy and effective solutions to teeth problems were found only during the 20th century. In 1950s, Brånemark used a titanium chamber to investigate blood circulation in rabbit bone, with plans to remove the chamber for re-use [2]. Unexpectedly, the titanium chamber was completely integrated into the bone without any sign of rejection. The concept of osseointegration was then born and defined since 29 May 1990 in the MeSH thesaurus (https://meshb.nlm.nih.gov/search, accessed on 1 June 2022) as: “The growth action of bone tissue as it assimilates surgically implanted devices or prostheses to be used as either replacement parts (e.g., hip) or as anchors (e.g., endosseous dental implants)”. Brånemark applied this concept to dental implants and, during the 1960′s–1970′s, implant innovation and designs expanded rapidly. Accordingly, implantology has made tremendous strides owing to its technology advances leading to high success rates.

The key requirement of any implantable material is biocompatibility with tissues and body fluids [3]. Biological compatibility of implants depends on their chemical composition and surface parameters such as topography, surface chemistry and charge, surface treatment, and physical/mechanical properties [4,5,6]. Titanium (Ti) and its alloys are among the best materials that exhibit excellent characteristics such as resistance to body fluid effects, great tensile strength, mechanical and anti-corrosion properties as well as good biocompatibility required for biomedical applications [7,8]. In this context, commercially pure titanium (TiCp) and titanium alloys, particularly Ti-6Al-4V, are regarded as “the gold standard” in implantology [9,10]. However, under different circumstances, Ti and its alloys show some limitations such as poor wear resistance in which the material wears out and produces some metallic particles and debris in the case of long-term implants. The released titanium particles and debris can cause local tissue lesions and inflammation at the implantation site, which ultimately induce implant failure [6,11,12,13]. To overcome these problems, mechanical and chemical properties have been improved and new titanium-based alloys were designed such as Ti-6Al-4V. Aluminum and vanadium contained in Ti-6Al-4V material both provide excellent mechanical strength. However, these materials may release their corresponding ions that can be toxic to the surrounding tissue, and therefore limit their clinical use. Indeed, previous studies have demonstrated that vanadium and aluminum exhibit cytotoxic effects and aluminum is involved in Alzheimer’s disease pathogenesis, bone fragility and local inflammation [13,14]. A recent study reported that nanostructures on the machined transgingival part of titanium implants may influence the surrounding tissue and require extensive preclinical basic research to gain clinical relevance [15]. Together, these observations suggest that the performance of titanium and titanium alloys as biomedical implants is inherently limited by the metal surface chemical, physical, and mechanical characteristics as well as the behavior of the biological host it interacts with. Therefore, the search for alloys that are free of toxic elements and inert in the oral environment is an increasingly critical requirement. Titanium-niobium alloys are used as an alternative to Ti-6Al-4V; however, little is known about their properties and behavior in the presence of human cells. Accordingly, we have undertaken this study to determine the in vitro biocompatibility of smooth titanium-niobium S_TiNb alloy compared to smooth TiCp (S_TiCp) discs using bone derived cell line (SaOs-2), assess the potential toxic effect of S_TiNb alloy on THP-1/macrophage cells as part of the first line of host defense, and study the potential toxic effect of potassium niobate (KNbO_3)_ salt on the transcriptome of THP-1 cells.

## 2. Materials and Methods

### 2.1. Preparation of Grade 4 and Niobium Titanium Discs

Grade 4 titanium (called “S_TiCp”) pellets of 1 cm diameter and 3 mm thickness were obtained from Champions-Implants GmbH (Flonheim, Germany). The niobium titanium containing 26% of Nb (TiNb) pellets of 1 cm diameter and 5 mm thickness were supplied by the Laboratoire d’Étude des Microstructures et de Mécanique des Matériaux (LEM3 UMR CNRS #7239, Metz, France). The smooth surfaces called “S” were obtained by polishing samples onto silica disc of adapted grain size. All samples were cleaned by sonication to remove remaining silica particles and sterilized following a “prion-free” sterilization program at 134 °C under 2.1 bar for a double 18 min-cycles.

### 2.2. Cell Culture

Human osteogenic sarcoma cells, SaOs-2, were obtained from the American Type Culture Collection (ATCC^®^ HTB85™), suspended in McCoy’s modified 5A medium (Gibco™, Paisley, UK) supplemented with 1% penicillin/streptomycin, 0.05% amphotericin B, and 15% fetal bovine serum (FBS, Sigma-Aldrich^®^, Saint Quentin Fallavier, France), and incubated in a humid atmosphere (37 °C, 5% CO_2_). THP-1 cells were obtained from the American Type Culture Collection (ATCC^®^ TIB202™) and grown in RPMI 1640 Medium (Gibco™, Paisley, UK) supplemented with: 2% L-glutamine, 1% penicillin/streptomycin, 0.05% amphotericin B, and 10% FBS.

### 2.3. Cell Viability Assay

Cell viability was assayed by two methods: (i) cell counting using Trypan blue (CAS #72-57-1, Bio-Rad Laboratories, Marnes-la-Coquette, France), and (ii) WST-1 assay™ (Roche Applied Science, Mannheim, Germany). For both assays, SaOs-2 cells were seeded in 12-well plates at a density of 1.6 × 10^4^ cells/well. THP-1 cells were seeded in 12-well plates at 3.0 × 10^5^ cells/well onto S_TiCp and S_TiNb discs or the original polystyrene dish. Both cell lines were cultured in humid atmosphere (37 °C, 5% CO_2_), in the presence of S_Ti and S_TiNb mirror-polished discs (Ra = 0.04 µm) for 24, 48, 72 and 96 h. At each time, the medium was discarded and the cells were removed by trypsin-EDTA (Sigma-Aldrich^®^, Saint Quentin Fallavier, France), washed twice and resuspended in fresh medium without FBS.

For the Trypan blue assay, a manual count (N = 4) was used to determine the average number of viable cells per well at 24, 48, 72 and 96 h following a Trypan blue exclusion staining method. The cell suspension was then plated in triplicate in 96-well plates and cultured in a humid atmosphere (37 °C, 5% CO_2_) during 24 h according to Nahle et al. [16]. With regard to the tetrazolium salt-reduction assay, 10 µL of WST-1 reagent were added to each well containing 100 µL of cell suspension and the cells were incubated for 4 h. Absorbance was measured at 450 nm using a spectrophotometer (FLUOStar Omega Microplate reader, BMG Labtech, Champigny-sur-Marne, France ). Finally, THP-1 cells were grown in increasing concentrations of KNbO_3_ salt in solution at: 0, 3.1, 6.25, 12.5, 25.0, 50.0, 100.0 and 200.0 µg/mL in RPMI medium for 24 h. Statistical analysis related to these experiments was performed using RLPlot 1.5.3. (Linux) using one-way ANOVA adjusted to Tukey-Kramer method (*p* value < 0.05).

### 2.4. Scanning Electron Microscopy

The morphology of SaOs-2 cells, seeded at 2 × 10^4^ cells/well was observed after a 72 h incubation period by SEM. The cells were fixed with 0.5% glutaraldehyde at 4 °C for 30 min, rinsed 3 times with phosphate buffer and then resuspended in 1% osmium for 30 min. The samples were then dehydrated for 15 min with an ascending ethanol gradient, 35%, 50%, 75%, 95%, 95%, 100% (*v/v*). Next, the samples were chemically dehydrated using hexamethyldisilane overnight in a vacuum desiccator. After complete drying, the samples were metallized for 5 min by gold sputtering to obtain a gold layer of ~15 nm and observed with the FEI Quanta 650 FEG™ SEM (FEI company, Hillsboro, OR, USA)).

### 2.5. Total RNA Extraction and Purification

Unexposed and exposed THP-1 cells to 25 µg/mL of KNbO_3_ salt (Sigma Aldrich, CAS # 12030-85-2, St. Louis, MO, USA) were grown at a final cell count of 1.5 × 10^6^ in quadruplicate in RPMI medium onto 20 mL in Greiner™ polystyrene dishes for 24 h. Cells were centrifuged to remove the remaining medium and membranes were destroyed with RLTplus™ buffer containing β-mercaptoethanol (Qiagen^®^, SAS, Courtaboeuf, France). Total RNA was extracted and purified using a RNeasy Plus Mini Kit (Qiagen^®^,#74136, SAS, France). RNA purity was assessed by spectrophotometry using a BioSpecNano^®^ (Shimadzu, Kyoto, Japan). RNA quality and integrity were assessed by microfluidic capillary electrophoresis using an RNA 6000 Nano Kit and Bioanalyzer™ 2100 (Agilent Technologies, Waldbronn, Germany). All RNA samples were of high purity and integrity, as demonstrated by A260/A280 ratios above 2, and RNA Integrity Numbers varying from 7.9 and 9.3. All RNA were stored at −80 °C until further use.

### 2.6. Microarray Hybridization

An aliquot (100 ng) of RNA from each sample was labeled with cyanine 3-CTP using Low Input Quick Amp Labeling kits (Agilent Technologies, Waldbronn, Germany). Labeled cRNAs were purified and hybridized onto Agilent G4851B SurePrint G3 Human Gene Expression 8 × 60 K v2 microarrays™ allowing a full coverage of the human transcriptome (Agilent Technologies, Waldbronn, Germany). Slides were washed and read by an Agilent G2505C™ microarray scanner with a 3 μm resolution and data were extracted using Agilent Feature Extraction software version 11.0 as described previously by Chézeau et al. [17]. The experiments (N = 4) were performed according to MIAME standards [18]. Microarray data have been uploaded to the NCBI Gene Expression Omnibus database [19], where they are accessible under GEO Series accession number GSE197987 (http://www.ncbi.nlm.nih.gov/geo/, accessed on 1 July 2022).

### 2.7. Statistical and Functional Analyses of Microarray Data

Data were quantile-normalized using Solo software (Institut de Génétique et de Biologie Moléculaire et Cellulaire, Strasbourg, http://www-microarrays.u-strasbg.fr/Solo/index.html, accessed on 1 May 2022). Genes displaying differential expression between control and exposed groups were identified using a method based on Fold Change Rank Ordering Statistics (FCROS; [20]). Genes for which Fold-Changes (FC) for exposed groups versus matched controls was at least 1.5 in either direction, and with f-values ≤ |0.01|, were considered as significantly differentially expressed. Details and functional information on genes differentially expressed were obtained using GeneCards™ database (https://www.genecards.org, accessed on 1 May 2022).

All transcripts displaying a relative fluorescence average (N = 4) higher than 109,000 relative units were considered for analysis. Gene-gene interactions were investigated using the Search Tool for the Retrieval of Interacting Genes/Proteins (STRING, https://string-db.org, accessed on 1 May 2022) database of physical and functional interactions v11.5 as described previously by Chézeau et al. [21]. The basic settings were: (i) full string network, (ii) meaning of network edge by evidence, (iii) involvement of all active interaction sources, and (iv) high confidence (0.7). Network nodes represent proteins and edges represent protein-protein associations [22]. A K-means clustering (K = 3) was also performed using the STRING software tool.

## 3. Results

### 3.1. Structural and Morphological Properties of Titanium Discs

**Figure 1:**
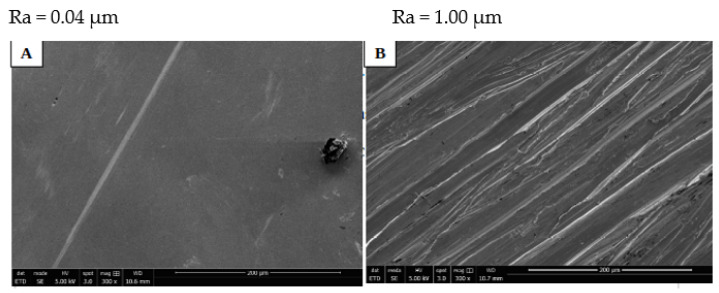
SEM images of: (**A**) mirror polished (S_TiNb) and (**B**) rough (R_TiCp) discs. Bars indicate the scale (similar magnification).

The morphology of S_TiNb discs without cells was analyzed using SEM. The mirror-polished surface of this material, shown in Figure 1A, was flat, regular, and smooth following mechanical polishing with an adapted to size grain silica disc. Furthermore, the average surface roughness (Ra) was of Ra~0.04, indicating that polishing was effective compared to the non-polished one, e.g., of R_TiCp which Ra was 1.00 µm (Figure 1B).

### 3.2. Proliferation and Metabolic Activity of SaOs-2 Cells

**Figure 2:**
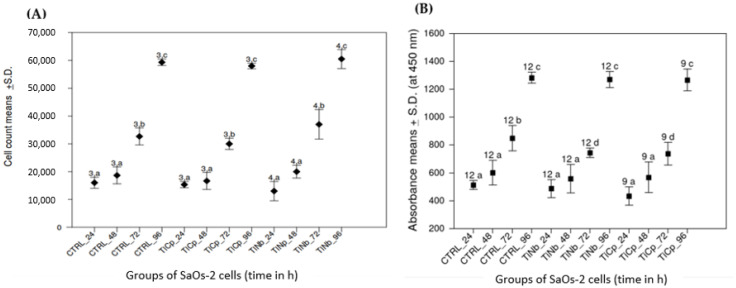
Grown (**A**) and metabolic activity (**B**) of SaOs-2 cells onto to S_TiCp and S_TiNb discs compared to CTRL polystyrene surface. Single-classification ANOVA. The number and the letter displayed onto the bars indicate, respectively, sampling replicates and statistical group. Groups not sharing the same letter are different on the 95% level (Tukey-Kramer method). Absorbance at 450 nm is expressed in mA; ‘CTRL’, ‘TiNb’ (S_TiNb) and ‘TiCp’ (S_TiCp) groups: growth on polystyrene, smooth TiNb and smooth TiCp alloys, respectively, for 24, 48, 72 and 96 h.

SaOs-2 cells were grown onto polished S_TiNb and S_TiCp discs compared to those cultured onto polystyrene surface (CTRL). Trypan blue excluding cells were counted at 24, 48, 72 and 96 h after seeding cells. We noticed that the proliferation of SaOs-2 cells was increased over time with no significant statistical difference when comparing cells growth onto S_TiCp and S_TiNb discs to the control (CTRL, Figure 2A). Moreover all 3 growth profiles (CTRL, S_TiCp and S_TiNb) were similar over the 96 h-growth period (*p* < 0.05, one-way ANOVA Tukey-Kramer method).

Metabolic activity of SaOs-2 cells was evaluated by measurement of mitochondrial dehydrogenase activity at 24, 48, 72 and 96 h of exposure to the biomaterials of interest. Similarly, an identical growth profile for CTRL, S_TiCp and S_TiNb was evidenced at 24 h and 48 h (*p* < 0.05, one-way ANOVA Tukey-Kramer method). However, a significant decrease of absorbance was noticed for cells growing onto S_TiCp and S_TiNb discs at 72 h compared to the CTRL; the observed relative decrease of metabolic activity was no longer evidenced at 96 h (*p* < 0.05, one-way ANOVA Tukey-Kramer method). Interestingly SaOs-2 cells showed the same growth profile on both S_TiCp and S_TiNb discs (Figure 2B).

### 3.3. Viability and Metabolic Activity of THP-1 Cells

Because macrophages are the primary phagocytes activated in the early inflammation stage when the body is exposed to implants [23], we used here THP-1 monocytes to investigate a potential toxicity of alloys or particles released from the degradation of dental implants, that could promote an activation of immune system. We compared the viability of THP-1 monocytes on polystryrene (CTRL) and S_TiNb surfaces.

**Figure 3:**
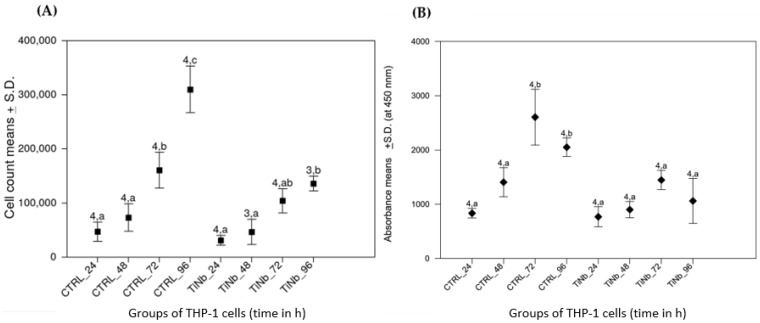
Grown (**A**) and metabolic activity (**B**) of THP-1 cells onto S_TiNb discs compared to CTRL polystyrene surface. The number and the letter displayed onto the bars indicate, respectively, sampling replicates and statistical group. Groups not sharing the same letter are different on the 95% level (Tukey–Kramer method). Absorbance at 450 nm is expressed in mA; ‘CTRL’ and ‘TiNb’ (S_TiNb) groups: growth on polystyrene and smooth TiNb alloy, respectively, for 24, 48, 72 and 96 h.

Figure 3A shows identical growth profiles of THP-1 cells onto either CTRL or S_TiNb surfaces during the first 48 h. Nevertheless, a significantly decrease of the viability as assayed by cell counting was noticed when cultured onto S_TiNb surface for 72 and 96 h compared to the control (Figure 3A).

Likewise, metabolic activity of THP-1 monocytes measured by succinate dehydrogenase activity was similar in both CTRL and S_TiNb groups during the first 48 h (Figure 3B). Beyond that, the metabolic activity reached a plateau then significantly decreased at 72 and 96 h in comparison to the CTRL group. Those results are in concordance with the data obtained from the cell counting (Figure 3A).

### 3.4. Morphology of SaOs-2 Cells Exposed to S_TiCp and S_TiNb Discs

**Figure 4:**
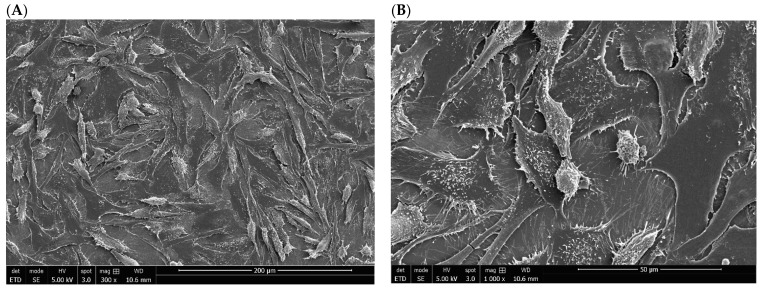
SEM image of SaOs-2 bone cells seeded on the surface of S_TiCp (mirror polished) disc at initial magnification of 300× (**A**) and 1000× (**B**) after a 72-h-incubation period.

The morphology and shape of human osteoblast-like cell line, SaOs-2, seeded on the on mirror polished of titanium materials were examined by SEM. Most of the SaOs-2 cells grown onto S_TiCp disc displayed an elongated and flat morphology; only a few of them had a round shape. They also formed a confluent layer and numerous pseudopodia, which are visible and well individualized at high magnification (×1000) and their organization seems to be random (Figure 4A,B).

**Figure 5:**
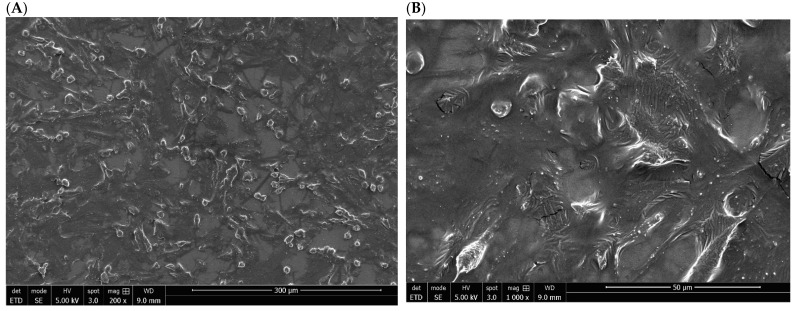
SEM image of SaOs-2 bone cells seeded on the surface of S_TiNb (mirror polished) disc at initial magnification of 300× (**A**) and 1000× (**B**) after a 72-h incubation period.

On the mirror-polished surface of S_TiNb discs, cultured SaOs-2 cells also displayed a random orientation and formed a nearly confluent layer. However, pseudopodia are less individualized, which could be due most likely to a less efficient gold sputtering metallization process (Figure 5A,B).

### 3.5. Influence of Increasing Doses of KNbO_3_ on THP-1 Cell Metabolic Activity

**Figure 6:**
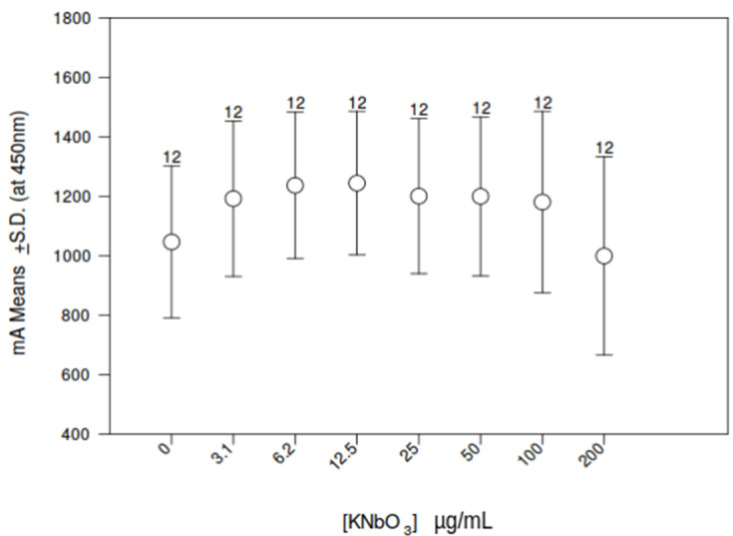
Metabolic activity of THP-1 cells exposed to various concentrations of KNbO_3_ determined by WST-1 assay.

Because little information is available about the immunotoxicity of dissolved niobium KNbO_3_, we exposed THP-1 monocytes to increasing concentrations of KNbO_3_ salt solution. Metabolic activity was examined after a 24 h period of incubation by measuring mitochondrial dehydrogenase activity using WST-1 assay. KNbO_3_ had no significant effect on the growth profile of THP-1 cells compared to the control group no matter the concentration (Figure 6). An hormesis effect, although not significant at the statistical level, was noticed when THP-1 monocytes were exposed to 3 to 50 µg/mL of potassium niobate. Therefore, we defined 25 µg/mL as an experimental dose for transcriptomic profiling.

### 3.6. THP-1 Transcriptome Analysis

As far as we know, no transcriptomic study has been undertaken so far on cells exposed to niobate anions. Therefore, we investigated the biological changes at the mRNA level of THP-1 cells exposed with KNbO_3_ salt. Transcriptome of THP-1 cells exposed to 25 µg/mL of KNbO_3_ solution during 24 h was determined using a FC ≥ |1.5| and a f-value ≤ |0.01| by comparison to unexposed cells both growing onto polystyrene surfaces. The analysis revealed only two differentially transcripts/62,976 probes dotted on the arrays. These transcripts are (i) *GPR156* that encodes a G protein-coupled receptors (GPCR) belonging to a large superfamily of cell surface receptors and that was slightly down-regulated (FC = 0.66 and f-value = 0.001), and (ii) *CTSZ* that encodes cathepsin Z protein, a lysosomal cysteine proteinase and member of the peptidase C1 family that was slightly up-regulated (FC = 1.52 and f-value = 0.998).

**Figure 7:**
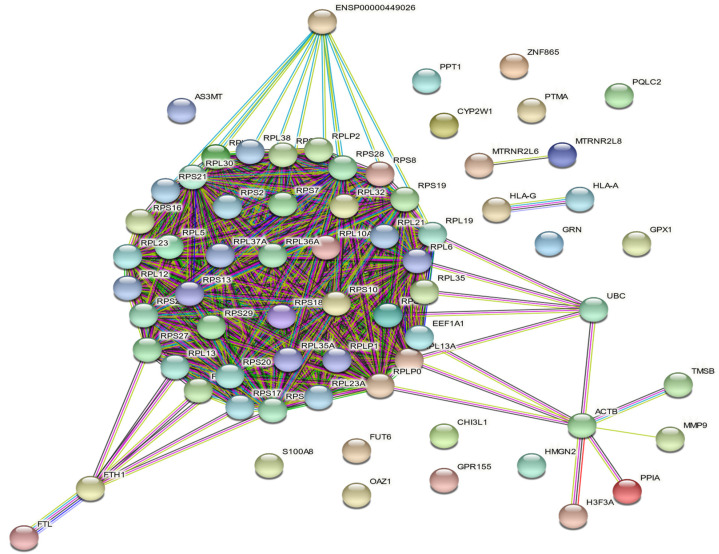
Network of gene-gene interactions in control and exposed SaOs-2 cells to 25 µg/mL of KNbO_3_ salt as analyzed by String database v11.5. Sixty-eight retrieved genes were plotted as they were recognized in String DB©. The line color of network edges indicates the type of gene interaction evidence by: (i) database in light blue line, (ii) experimental in purple line, (iii) neighborhood in green line, (iv) fusion in red line (evidence), (v) co-occurrence in blue line, (vi) text mining in yellow line, (vii) black line (co-expression evidence) and, finally, (viii) protein homology in violet.

As the transcriptomes of THP-1 monocytes in presence and in the absence of potassium niobate have shown high similarities in gene-expression profiles, we retrieved transcripts displaying relative fluorescence above 109,000 relative units, namely 114 dots that corresponded to 87 genes after removing redundancy. Of them, 85 were coexpressed in both samples (Table 1). Sixty-eight among the 85 common series were recognized as human proteins and plotted (Figure 7) by the STRING database v11.5. Seventeen transcripts were not recognized by Ensembl: (i) the A_XX series (10 transcripts) as they are specific to the used array, (ii) one X_LOC not recognized by Ensembl, (iii) *FAM74A4*, *RNA28S5*, *SNAR-A3, SNAR-B2, SNAR-D* and *THC2526015* (Table 1).

At the chosen level of expression, 4 transcripts were not found in the KNbO_3_ exposed series, which are *Ribosomal ProteinL11 (RPL11), Ribosomal Protein L7a (RPL7A), Ribosomal Protein S20 Pseudogene 27 (RPS20P27)* and *Small NF90 (ILF3) Associated RNA D (SNAR-D)*. These 4 transcripts were expressed at levels below 109,000 relative units. However, 81/85 transcripts (slightly more than 95%), were all co-expressed at a high level of fluorescence in both series and presented in Table 1.

A K-means clustering (K = 3) determined (Figure 7):

- (i) a first cluster of 46 transcripts that encode cytosolic proteins figured as red nodes: *AS3MT*, *ENSP00000449026*, *FTH1*, *FTL*, *FUT6*, *RPL10A*, *RPL12*, *RPL13*, *RPL13A*, *RPL19*, *RPL21*, *RPL23*, *RPL23A*, *RPL30*, *RPL32*, *RPL35*, *RPL35A*, *RPL36A*, *RPL37A*, *RPL38*, *RPL5*, *RPL6*, *RPL7A*, *RPLP0*, *RPLP1*, *RPLP2*, *RPS10*, *RPS11*, *RPS13*, *RPS16*, *RPS17*, *RPS18*, *RPS19*, *RPS2*, *RPS20*, *RPS21*, *RPS25*, *RPS27*, *RPS28*, *RPS29*, *RPS3A*, *RPS5*, *RPS6*, *RPS7*, *RPS8*, *S100A8* transcripts that encode proteins mainly involved in biological process ‘GO:0000184—nuclear-transcribed mRNA catabolic process’ (41/46 counts),
- (ii) a second cluster of 11 transcripts figured by green nodes: *ACTB*, *CHI3L1*, *EEF1A1*, *GPR155*, *H3F3A*, *HMGN2*, *MMP9*, *OAZ1*, *PPIA*, *TMSB4X*, *UBC* of which a functional enrichment was the cellular component ‘GO:1904813—ficolin-1-rich granule lumen’ (3/11 counts),
- and finally (iii) 11 transcripts of cluster 3 colored in blue: *CYP2W1*, *GPX1*, *GRN*, *HLA-A*, *HLA-G*, *MTRNR2L6*, *MTRNR2L8*, *PPT1*, *PQLC2*, *PTMA*, *ZNF865* corresponding to the biological process ‘GO:0042270—protection from natural killer cell mediated cytotoxicity’ (2/11 counts).

## 4. Discussion

Despite the actual innovation in dental implantology, there remain challenges with the development of adequate biomaterials for clinical applications. Indeed, some patients can still suffer from complications due to the selection, design, and manufacturing of implant biomaterial [24]. Important metal selection problems can result from the interaction between implant alloys and targeted tissue and their adverse event on host health. Hence, adequate biocompatibility sounds to be one of the key benchmarks for metal biomaterials. Among the various materials currently in use, titanium alloys are rapidly emerging as the first choice for most applications [25]. However, the impact of their characteristics on the biocompatibility of host tissues remains an issue. Therefore, the present study was designed to evaluate in vitro the biocompatibility of S_TiNb versus commercially pure S_TiCp using bone-derived and immune-cell lines and assess the potential immunotoxicity of KNbO_3_ salt on immune cells by transcriptome analysis. Our main finding revealed that S_TiCp and S_TiNb discs have no significant impact on the proliferation and viability of SaOs-2 cell model, whereas, a significant decrease in THP-1 cells proliferation and viability was observed in the presence of S_TiNb discs. Importantly, no significant changes were found neither at the metabolic activity nor at the transcriptomic levels of THP-1 monocytes exposed to KNbO_3_ salt suggesting that potentially released niobium nanoparticles may not have an effect on the immune system.

To determine the biocompatibility of titanium-niobium alloy, we first performed all our experiments on smooth discs because we noticed that rough surfaces of TiCp influence the direction of SaOs-2 cells proliferation (data not shown). Precisely, cells grown on smooth surfaces proliferate in all directions contrary to those grown on rough surfaces of TiCp. Interestingly, previous studies have shown a tendency towards more bone loss and higher incidence of implant loss around rougher surface implants [26,27]. In contrast, other studies reported that rough-surfaced implants promote osseointegration favoring both bone anchoring and bio-mechanical stability, [28,29]. These controversies could be due to the heterogeneity and variability in the study design as well as the effect of unknown confounding factors, which make it difficult to draw definitive conclusions.

Findings from our in vitro experiments showed clearly that the surface structure and the composition of S_TiNb discs, as well as S-TiCp discs, have no effect on cellular attachment, adherence, proliferation, and survival of SaOs-2 cells as compared to polystyrene surfaces, implying that smooth titanium-niobium alloys may be used safely in dental implants without complications. These results are consistent with findings reported earlier with respect to the compatibility of S_TiCp [9,25,30,31,32]. Recent studies have highlighted the biocompatibility of titanium-niobium alloys on various cell lines such as fibroblasts, osteoblasts, mesenchymal stem cells [33,34,35,36]. Other investigations have reported that the use of a Ti–26Nb (at.%) alloy not only reduces the Young module, but reveals high biocompatibility on osteoblast cells, comparable to control or cells growing onto TiCp [37,38]. Because niobate ions are less toxic than the vanadate ones and niobium-based alloys promote cell-to-cell interactions, niobium may be used as alternative material to vanadium in the design of future titanium alloys [10,14,37,38,39].

To our surprise, a significant decrease in THP-1 viability and metabolic activity at 72 h and 96 h of exposure to S-TiNb was noticed in our experiments. Interestingly, this result was neither evidenced in the present study using SaOs-2 cells nor previously reported in literature [34]. One possible explanation for this observation is that the decrease in cells’ viability could be due to a cell-line effect on the biomaterial, which is followed by niobate leakage, since macrophages are known to have the ability to erode mineral surfaces making them prone to leakage as it was reported previously [40,41]. To the best of our knowledge, no biocompatibility study was previously conducted using monocyte/macrophage cells model grown onto TiNb, while this cell line was already used for biocompatibility studies on TiCp surfaces [9,42,43,44].

As immune cells, especially macrophages and dendritic cells—the first and the main effectors of immune system—are involved in osseointegration failure of implants [23], our main objective was to analyze the grown potential of THP-1 cells expected more sensitive to metal leakage in presence of a wide range of niobate ion doses (KNbO_3_ salt): 3.1 to 200 μg/mL. Indeed, released niobium species may induce local inflammation, attract bacteria and, finally, induce biofilm formation onto TiNb implants, as was evidenced with TiCp [45]. Finally, osteolysis may occur that leads to dental peri-implantitis, the main cause of implant failure. In this study, no significant changes were found at the metabolic activity of THP-1 monocytes exposed to KNbO_3_ salt, suggesting that niobium nanoparticles have no deleterious effect on cell proliferation and no apparent toxicity (Figure 6). A similar result was obtained on SaOs-2 cells (data not shown).

In support to our findings, previous studies have demonstrated that niobium, tantalum, zirconium, and molybdenum, when incorporated into titanium-based alloys, do not present any cytotoxic reactions on different type of cells [10,46,47]. Moreover, KNbO_3_ nanoparticles displayed a combination of low toxicity and nonlinear optical properties, which make them attractive for use as a bio-imaging material [48].

The fact that KNbO_3_ salt does not impact viability of THP-1 cells caused us to question whether it causes changes in these cells at the molecular level. To address this concern, we tested the effect of niobate potassium on the whole transcriptome of THP-1, since potential immunotoxicity was previously studied at the transcriptomic or proteomic level by analyzing only few selected genes products involved in either growth pattern or inflammatory response without conducting experimentation at the whole transcriptome [42,43,44,49]. Here, transcriptomic analysis was conducted for the first time to our best knowledge, by growing THP-1 monocytes in the presence of 25 μg/mL KNbO_3_ after 24 h-period of exposure. We investigated a 24 h-incubation period, because the potential effect of KNbO_3_ salt on gene transcription should appear more quickly compared to the grown of THP-1 onto S_TiNb discs. Even if a significant decrease in proliferation occurred only after a 48 h-exposure period (Figure 3A,B), the potential Nb leaching process and its eventual internalization into cell being time dependent should occur from a few hours of contact. Our transcriptomic analysis revealed two out of a total of 62,976 probes were slightly differentially expressed and identified as *GPR156* and *CTSZ*. *GPR156* encodes G-protein-coupled receptors (GPCRs), a large superfamily of cell surface receptors characterized by seven helical transmembrane domains, together with N-terminal extracellular and C-terminal intracellular domains. This transcript is poorly studied in literature and is unrelated to dental implants. *CTSZ* encodes the cathepsin Z, a catalytic function protein strongly involved in carboxy-monopeptidase and carboxy-dipeptidase reactions. This protein is found in both osteoblast and osteoclast lineages, and was proposed as a novel potential biomarker for osteoporosis [50]. Nevertheless, slight dysregulation of only one isolated transcript may have poor biological significance as protein performs its function as a part of a biological processes through and within a network of other molecules.

However, it is striking to show that 95% of the most-expressed genes were similar in both groups: CTRL, and cells exposed to potassium niobate, indicating that even in the case of leakage of niobate, there was no significant effect on normal growth and metabolic activity of THP-1 cells (Table 1). The same three clusters of the most-expressed genes were shown in each group, mainly genes involved in ribosome machinery and mRNA translation, genes indicating an active growth of cells. In addition, our analysis did not find any differentially expressed metalloprotein genes known to recruit Fe^++^, Cd^+^, Cu^++^, Zn^2^+ or anion transporter proteins following THP-1 exposure to potassium niobate in contrast to data obtained earlier following exposure of Zn nanoparticles to the same THP-1 monocytes [51]. No genes involved in necrosis/apoptosis or autophagy were overexpressed in THP-1 monocytes exposed to potassium niobate in contrast to THP-1 exposed cells to 50 µg/mL of polymeric nanoparticles, indicating a clear lack of immunotoxicity [52].

With regard to TiNb alloy, a previous study performed on human osteoblatic cells grown on TiNb did not show any significant variation in selected markers of bone cells including *transforming growth factor-β*, *integrin-β1*, *alkaline phosphatase*, *osteopontin*, *macrophage colony stimulating factor*, *prostaglandin E synthase*, and *apolipoprotein E* [53]. Deposition of a niobium/zirconium/tantalum alloy on the surface of Ti-6Al-4V had shown unchanged expression of the *alkaline phosphatase* gene (*ALPL*) in human osteosarcoma cells (MG-63). Additionally, the surface treatment with Nb promotes better performance of L-929 mouse cells attachment to the modified surface as evidenced by higher RT-QPCR rates of *type I α1-collagen* and *fibronectin 1*, ensuring earlier osteogenic differentiation [54]. A biocompatibility study performed on osteogenic cells from newborn rat calvaria with a G4-Ti-35 niobium alloy did not reveal any change in the following bone biomarkers: *alkaline phosphatase*, *runx-2*, *osteocalcin* and *osteopontin* [55]. Taken together, our transcriptomic study suggests an absence of immunotoxicity of soluble KNbO_3_ on THP-1 cells. We speculate that potential toxicity of S_TiNb on THP-1 was not related to niobium leakage, but to the manufacturing process of TiNb alloy. Another thesis is that toxicity could be related to the size of the particles released around implants, even if a study conducted on KNbO_3_ nanoparticles highlighted low toxicity [48]. However, further experiments are necessary to validate these hypotheses.

To summarize, our study reports that commercially S_TiCp and S_TiNb alloy have no significant adverse effects on the proliferation and viability of SaOs-2 cell model, whereas, a significant decrease in THP-1 cells proliferation and viability was observed in the presence of S_TiNb discs. Subsequent experiments exploring the impact of KNbO_3_ nanoparticles on THP-1 monocytes metabolic activity and transcriptomic profiling support the hypothesis that the latter decrease may not be related to niobium leakage but to the manufacturing process of TiNb alloy. Without doubt, more research is needed into the biocompatibility of titanium alloys using state-of-the art manufacturing technologies available today along with in vivo and clinical studies.

## Figures and Tables

**Table 1:** Common genes expressed in CTRL and KNbO_3_ salt exposed THP-1 cells. *: match mainly exon flanks boundaries and are external name to Ensembl GRCh37© release 105—Dec 2021 (named following Array SurePrint_G3_GE_8 × 60 k; Human AGILENT Probe™). The following genes co-expressed at high level were not considered by String database: *FAM74A4*, *RNA28S5*, *SNAR-A3*, *SNAR-B2*, *SNAR-D* and *THC2526015*.

*A_23_P336854*	Human Agilent Probe/RPL12L3-001 *
*A_24_P204144*	Human Agilent Probe/Not recognized by Ensembl^©^
*A_33_P3270581*	Human Agilent Probe/RP11-79L9.2-001 *
*A_33_P3299436*	Human Agilent Probe/RP5-827C21.1-001 *
*A_33_P3315027*	Human Agilent Probe/RP11-510I6.1-001 *
*A_33_P3353259*	Human Agilent Probe/RP11-179H18.5-001 *
*A_33_P3370515*	Human Agilent Probe/Not recognized by Ensembl^©^
*A_33_P3375668*	Human Agilent Probe/AP000354.2-001 *
*A_33_P3381429*	Human Agilent Probe/RP11-16L9.1-001*
*A_33_P3396434*	Human Agilent Probe/No external name
*ACTB*	Actin, cytoplasmic 1
*AS3MT*	Arsenite methyltransferase
*CHI3L1*	Chitinase-3-like protein 1
*CYP2W1*	Cytochrome P450 2W1
*EEF1A1*	Elongation factor 1-alpha 1
*FTH1*	Ferritin heavy chain
*FTL*	Ferritin light chain
*FUT6*	Alpha-(1,3)-fucosyltransferase 6
*GPR155*	Integral membrane protein GPR155
*GPX1*	Glutathione peroxidase 1
*GRN*	Granulin precursor
*H3F3A*	H3 histone family member 3A
*HLA-A*	HLA class I histocompatibility antigen
*HLA-G*	HLA class I histocompatibility antigen, alpha chain G
*HMGN2*	Non-histone chromosomal protein HMG-17
*MMP9*	Matrix metalloproteinase-9
*MTRNR2L8*	MT-RNR2 like 8
*MTRNR2L6*	Humanin-like 6
*OAZ1*	Ornithine decarboxylase antizyme 1
*PPIA*	Peptidyl-prolyl cis-trans isomerase A;
*PPT1*	Palmitoyl-protein thioesterase 1
*PQLC2*	Solute carrier family 66 (lysosomal lysine-arginine transporter)
*PTMA*	Prothymosin alpha
*RPL5*	60S ribosomal protein L5
*RPL6*	60S ribosomal protein L6
*RPL7A*	Large subunit ribosomal protein L7ae
*RPL10A*	60S ribosomal protein L10a
*RPL12*	Large subunit ribosomal protein L12e
*RPL13*	Large subunit ribosomal protein L13e
*RPL13A*	Large subunit ribosomal protein L13ae
*RPL19*	Large subunit ribosomal protein L19e
*RPL21*	60S ribosomal protein L21
*RPL23*	Large subunit ribosomal protein L23e
*RPL23A*	60S ribosomal protein L23a
*RPL30*	Large subunit ribosomal protein L30e
*RPL32*	Large subunit ribosomal protein L32e
*RPL35*	60S ribosomal protein L35
*RPL35A*	Large subunit ribosomal protein L35ae
*RPL38*	Large subunit ribosomal protein L38e
*RPL41*	Ribosomal protein L41
*RPLP0*	Ribosomal protein lateral stalk subunit p0
*RPLP1*	60S acidic ribosomal protein P1
*RPLP2*	60S acidic ribosomal protein P2
*RPS2*	Small subunit ribosomal protein S2e
*RPS3A*	40S ribosomal protein S3a
*RPS5*	Small subunit ribosomal protein S5e
*RPS6*	40S ribosomal protein S6
*RPS7*	40S ribosomal protein S7
*RPS8*	Small subunit ribosomal protein S8e
*RPS10*	40S ribosomal protein S10
*RPS11*	Small subunit ribosomal protein S11e
*RPS13*	Small subunit ribosomal protein S13e
*RPS16*	Small subunit ribosomal protein S16e
*RPS17*	Small subunit ribosomal protein S17e
*RPS18*	40S ribosomal protein S18
*RPS19*	40S ribosomal protein S19
*RPS20*	Small subunit ribosomal protein S20e
*RPS21*	Small subunit ribosomal protein S21e
*RPS25*	Small subunit ribosomal protein S25e
*RPS27*	40S ribosomal protein S27
*RPS28*	Small subunit ribosomal protein S28e
*RPS29*	Small subunit ribosomal protein S29e
*S100A8*	Protein S100-A8
*TMSB4X*	Thymosin beta 4, X-linked
*UBC*	Polyubiquitin-C
*XLOC_014512*	RP11-54O7.1 (Clone-based (Vega) gene)
*ZNF865*	Zinc finger protein 865

## Data Availability

Microarray data have been uploaded to the NCBI Gene Expression Omnibus database where they are accessible under GEO Series accession number GSE197987.
